# Preliminary Experience on Laser Interstitial Thermal Ablation Therapy in the Treatment of Extra-axial Masses: Indications, Imaging Characterization and Outcomes

**DOI:** 10.7759/cureus.2894

**Published:** 2018-06-29

**Authors:** Armando Ruiz, Roberto J Diaz, Simon Buttrick, Michael Ivan, Mehul Desai, Ricardo J Komotar, Rostislav Medvid

**Affiliations:** 1 Radiology, University of Miami, Miami, USA; 2 Neurology and Neurosurgery, Montreal Neurological Institute and Hospital, Montreal, CAN; 3 Department of Neurosurgery, University of Miami Miller School of Medicine, Miami, USA; 4 Radiology, University of Miami Miller School of Medicine, Miami, USA; 5 Neurological Surgery, University of Miami Miller School of Medicine, Miami, USA; 6 Radiology, University of Miami, MIAMI, USA

**Keywords:** meningioma, fibrous tumor of the dura, laser interstitial thermal therapy, extra-axial brain masses, intra-axial masses

## Abstract

Laser thermal ablation is a novel minimally invasive neurosurgical technique that has proven to be beneficial in the treatment of a select group of neurosurgical conditions such as primary brain neoplasms, brain metastases, radiation necrosis, and epileptogenic lesions such as cortical dysplasia and mesial temporal sclerosis.

The applicability of laser thermal ablation and its utility in the treatment of extra-axial (EA) brain neoplasms, mainly meningioma, is another novel use of this technique. Our article discusses the use and benefits of this technique in this particular clinical scenario. We describe our experience in a group of symptomatic patients from our institution with EA masses, mainly recurrent meningiomas, that failed previous more conventional treatment therapies such as surgery and radiotherapy. Our paper emphasizes patient selection, indications for the procedure, and post-treatment imaging characteristics of the ablated lesions.

## Introduction

Magnetic resonance imaging (MRI) guided laser interstitial thermal therapy (LITT) is a minimally invasive technique, which delivers light energy to target tissue resulting in carefully controlled highly specific thermal ablation and minimal collateral damage. In neuro-oncology, LITT is primarily used as salvage therapy for recurrent intra-axial (IA) masses such as gliomas, metastatic disease and radiation necrosis [1]. One particularly important property of the technique is that it can be safely administered to patients with a history of maximal irradiation and multiple prior craniectomies who are otherwise at increased risk of wound breakdown if new surgery is elected.

Until recently, little data existed on the utility of LITT in the management of extra-axial (EA) masses. In 2016, Tatsui et al. demonstrated the safety and efficacy of LITT in the treatment of selected cases of metastatic epidural spinal cord compression [2]. Kato et al. first described the use of stereotactic thermal ablation of the base of skull meningiomas using radiofrequency probes [3-4]. Subsequently in 2012, Jethwa et al. first used LITT to ablate intracranial meningiomas, but did not provide long term follow-up data or imaging details [5]. A recent case series by Ivan et al. described their experience in five patients with LITT ablated recurrent intracranial EA tumors [6]. The authors reported no perioperative complications, satisfactory long-term local control in three low-grade meningiomas, but disease recurrence and progression in two high-grade meningeal neoplasms. Our article uses the same cohort of five patients and focuses on the post-ablation imaging characteristics, temporal evolution and variable responses to treatment of recurrent intracranial EA tumors treated with LITT.

## Technical report

Materials and methods

Five patients underwent LITT of recurrent EA dural based supratentorial lesions located in the parasagittal or lateral convexity regions (Table 1). Indication for treatment included radiologic progression of tumor with or without new symptoms despite several prior surgical resections and radiation therapy with maximal radiation doses already achieved (Table 1). All patients were deemed poor surgical candidates due to increased risk of wound breakdown and poor wound healing. Single catheter trajectory LITT (Medtronic, Minneapolis, USA) was used as minimally invasive salvage therapy for treatment-refractory recurrent disease as described by Ivan et al. [6].

**Table 1:** Patients characteristics and outcomes F: frontal; L: left; P: parietal; R: right; SFT: solitary fibrous tumor; LITT: laser interstitial thermal therapy; WHO: World Health Organization; N/A: not applicable.

case	sex	age	tumor	grade	location	length of imaging follow up in months	post-LITT residual tumor	local tumor control	disease progression	time to progress	pre-LITT symptoms	post LITT symptoms at one month	
1	F	65	Meningioma	WHO I	R F parafalcine	9	yes	yes	no	N/A	none	none	
2	M	67	Meningioma	WHO I	L F parasagittal	7	yes	yes	no	N/A	headache	none	
3	M	67	Meningioma	WHO III	R FP parasagittal	10	yes	no	yes	3 months	none	none	
4	F	82	Meningioma	WHO I	L F parasagittal	10	yes	yes	no	N/A	none	none	
5	M	45	SFT	N/A	L F convexity	23	yes	no	yes	10 months	seizures	none	

Pre-treatment radiographic progression was defined as persistent lesion growth on two serial preprocedural MRI studies. Changes in pre-treatment tumor volume, as measured on 1 mm cut T1 three‐dimensional magnetization‐prepared rapid gradient‐echo imaging (3D MP RAGE), contrast-enhanced images, were used to calculate the pre-operative volumetric growth rate of each lesion. Twenty-four hours prior to LITT, all patients underwent pre-operative MRI with and without contrast with neuronavigation protocol. On the day of the procedure, ablation was monitored with realtime MRI thermal imaging [1]. Ablation volume estimates were displayed using Medtronic software.

Each patient underwent two intra-procedural T1 contrast-enhanced scans: the first scan was performed prior to ablation to define the lesion borders while the second scan occurred during and immediately after LITT to evaluate ablation efficacy. A complete postprocedural brain MRI with and without contrast was performed twenty-four hours after LITT to better characterize the ablated lesions, to evaluate for post-surgical complications, and to serve as a baseline for all future follow up studies. Follow up imaging with contrast-enhanced MRI was performed at one-month post-LITT and at additional selected time points until the patient was lost to follow up or required no further follow up.

Pre-LITT, post-LITT and post-procedural follow up MRI’s were retrospectively evaluated for changes in imaging characteristics within and around each treated lesion. Examined intralesional characteristics include changes in lesion size, enhancement pattern, and intralesional T1, T2 and fluid-attenuated inversion recovery (FLAIR) signal.

Extralesional characterstics include perilesional FLAIR signal, mass effect, midline shift and any other incidentally noted signal changes. Residual disease was defined as the presence of residual nodular enhancement on 24 hours post-LITT MRI. Satisfactory local tumor control was defined as the decrease or stability of post-LITT tumor volume and lack of enhancement of lesion core on all subsequent follow-up imaging. Disease progression was defined as >10% increase in post-LITT tumor volume on serial post-procedural follow-up imaging.

Results

Five patients with recurrent EA dural-based lesions underwent treatment with LITT: three patients with World Health Organization I (WHO) meningioma, one patient with WHO III meningioma and one patient with solitary fibrous tumor (SFT) (Table 1). Average treated tumor volume was 29.7 cc with a maximal diameter of 3.5 cm. No intra or post-procedural complications occurred. Two of five patients who were symptomatic before LITT reported resolution of symptoms after ablation. Immediately after ablation, all patients demonstrated imaging evidence of residual mass. Comparison of twenty-four hours pre and post-LITT images (Table 2) demonstrated a post-ablation nonenhancing core with a trend toward increased T1 and decreased T2 central intralesional signal. All ablated lesions demonstrated a rim of peripheral enhancement. No significant change in lesion size was noted on twenty-four hours post-LITT images. Two of five patients had minimal increase or development of new minimal perilesional vasogenic brain edema. Two of five patients demonstrated increased T1 perilesional brain signal on twenty-four hours post-LITT MRI prior to intravenous (IV) contrast administration. The edema and the perilesional signal changes were not associated to new neurological symptoms.

Post-procedural imaging follow-up ranged from seven to twenty-three months. All patients with a diagnosis of WHO I meningioma demonstrated satisfactory local tumor control: the central non-enhancing portion continued to decrease in size, while the enhancing periphery collapsed around it. The residual disease manifested as a focal non-enhancing nodule with a peripheral enhancing rim. In some cases, the residual area of focal asymmetric enhancement increased in prominence on follow up, but the overall size of the ablated lesion core remained stable or continued to decrease. The remaining two patients with diagnoses of WHO III meningioma and SFT demonstrated imaging signs of disease progression. Case 3 with WHO III fulfilled imaging criteria for disease progression three months after LITT. Case 5 with SFT showed satisfactory post-LITT tumor control during the first eight months following ablation (with a resolution of pre-LITT symptoms), followed by a subsequent progressive increase in tumor size first noted on 10-month follow-up. This case fulfilled imaging criteria for disease progression eleven months after LITT accompanied by recurrence of facial numbness.

**Table 2:** Magnetic resonance imaging (MRI) characteristics of ablated extra-axial (EA) laser interstitial thermal therapy (LITT) lesions Iso: isointense; homo: homogeneous enhancement; hetero: heterogeneous enhancement; periph: peripheral enhancement; FLAIR: fluid-attenuated inversion recovery +C=Contrast. ↑ minimally increased ↓ hypointense ± heterogeneously hyperintense *edema = vasogenic

case	T1	T1	T1 +C	T1+C	T2	T2	FLAIR	FLAIR	edema	edema	perilesional high T1
	pre	24 h	pre	24 h	pre	24 h	pre	24 h	pre	24 h	
1	iso	no change	homo	periph	±	no change	±	no change	no change	no change	no
2	iso	↑	homo	periph	±	↓	iso	↓	none	↑	yes
3	↓	↑	homo	periph	iso	↓	iso	↓	mild	no change	no
4	iso	↑	homo	periph	±	↓	↑	↓	mild	no change	yes
5	↓	↑	homo	periph	↑	no change	iso	no change	mild	no change	no

## Discussion

The purpose of this article is to describe the imaging characteristics of EA masses following treatment with LITT and to highlight the similarities and differences between ablated EA and IA lesions and to establish imaging criteria to define success vs. treatment failure.

**Figure 1:**
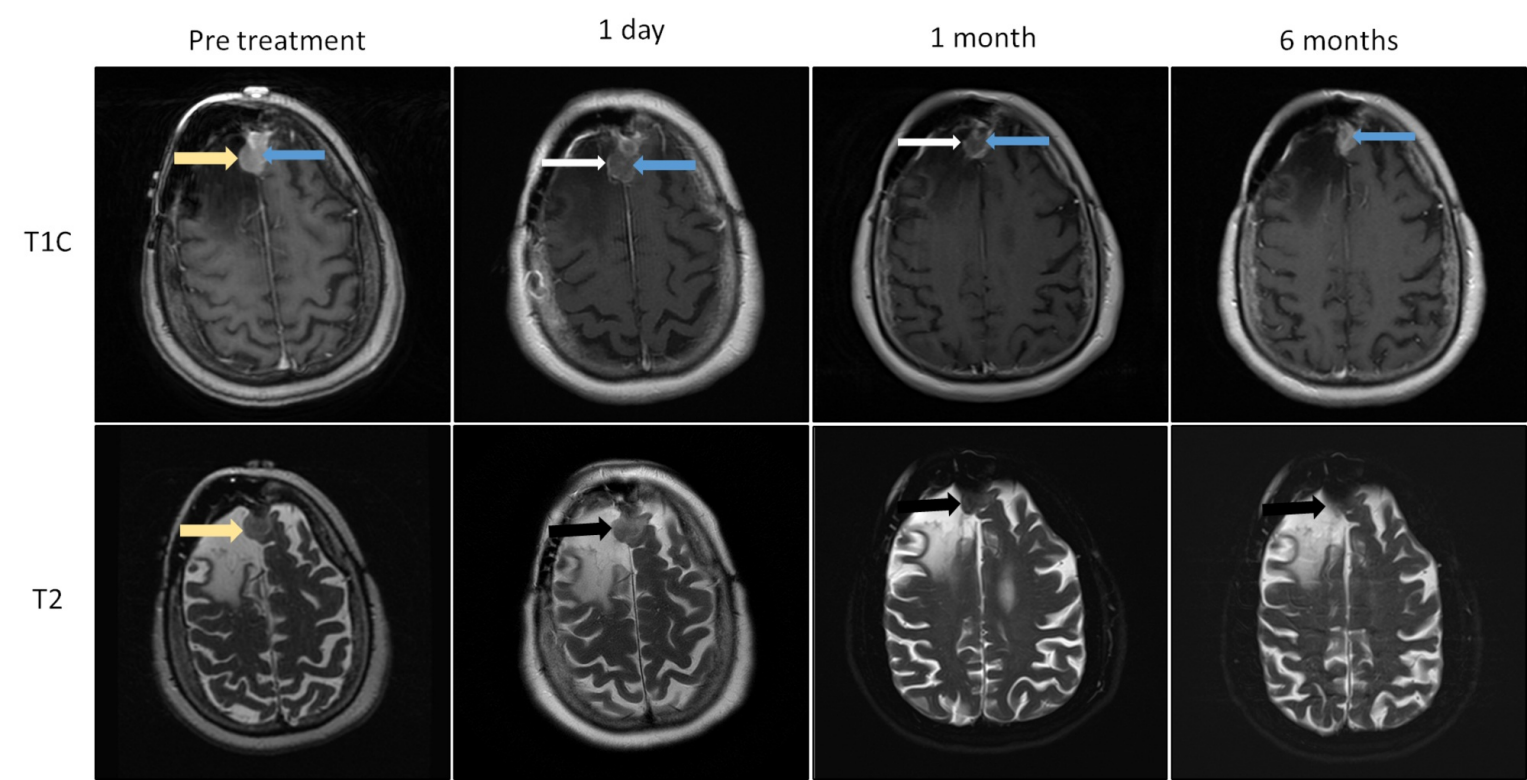
Normal evolution of extra-axial (EA) laser interstitial thermal therapy (LITT) ablated mass WHO I meningioma; (case one) Images obtained the day before LITT, one day, one month and six months following LITT. The pre-treatment image demonstrates an enhancing parafalcine mass (yellow arrow). In the ablated extra-axial masses, the central core is composed of a non-enhancing central zone of coagulation necrosis (vanishing enhancement sign), (blue arrow), bounded by a thin peripherally enhancing rim (white arrow). The mass remains stable in size with low T2 signal immediately after ablation (black arrow), followed by progressive decrease thereafter (one month and six months). WHO: World Health Organization.

**Figure 2:**
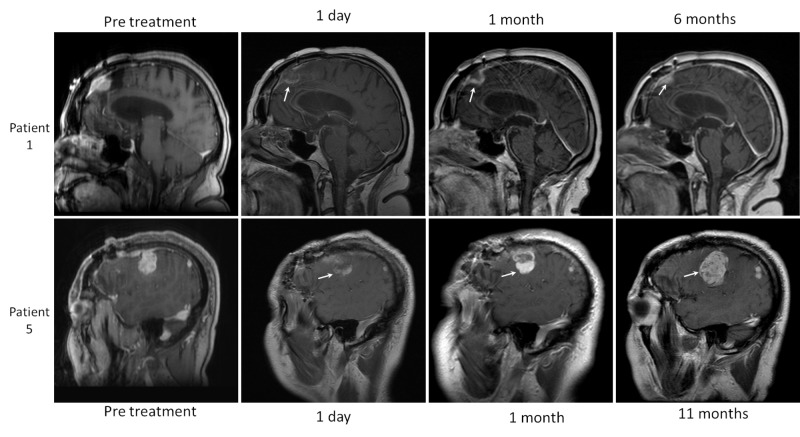
Evolution of extra-axial (EA) laser interstitial thermal therapy (LITT) treated masses Residual mass was noted in all ablated extra-axial lesions presenting with peripheral enhancing margins (arrows). All World Health Organization I meningiomas (WHO I), demonstrated satisfactory local tumor control after ablation marked by an overall decrease in lesion size (white arrow patient 1). Patients with high-grade lesions (white arrow patient 5), demonstrated rapid growth consistent with disease progression of residual tumor usually starting at the periphery of the mass.

IA lesions increase in size immediately after ablation due to development of a peripheral zone of intralesional edema and delayed liquefaction necrosis [1,7], which may continue to increase in size over the first 1-40 days [8]. The central zone of the ablated core generally demonstrates increased T1 and decreased T2 signal related to the presence of subacute blood products and coagulation necrosis [1,7]. In contrast, EA lesions (meningiomas, fibrous tumor of the dura), do not change in size after ablation perhaps due to their more compact fibrous nature (Figure 1). Other notable differences in the post-LITT appearance are the lack of lesional enhancement (vanishing enhancement sign), and the relative lack or minimal increase of perilesional vasogenic brain edema immediately and twenty-four hours after ablation in EA compared to IA lesions. In our EA cohort, three of five lesions developed minimal post-procedural vasogenic edema, while the remaining two lesions showed no extralesional change in signal (Figure 1). In contrast, all IA masses demonstrate significant post-LITT perilesional edema, which reaches maximum dimensions at one to three days, followed by a gradual decrease in size over the next two to eight weeks [1]. Due to concerns over intractable intracranial hypertension [9] Jethwa et al. have recommended that IA lesions over 3 cm in size should undergo staged ablation over multiple sessions [10]. This concept may not apply to all type of EA lesions. If our findings are corroborated, one potential implication is that single session multicatheter ablations of larger meningiomas and fibrous tumors may be a safe option. The imaging differences between ablated EA and intraparenchymal lesions are likely related to inherent differences in their optical and thermal properties. Eggert et al. found no statistically significant differences between the optical properties of 17 meningiomas and 13 gliomas. Findings were attributed to low sample size and high statistical dispersion of the measured physical values [11]. Our study suggests that differences in optical properties between IA and EA lesions may indeed exist. Currently, real-time quantitative estimates of ablation during intracranial LITT are calculated by using the known optical properties of gliomas irrespective of tumor type. If significant differences in optical properties do indeed exist, then the current approach may be a source of error in intra-operative real-time ablation zone estimates for non-glial tumors, leading to suboptimal ablation at tumors’ periphery. A potential long-term research aim would be the creation of libraries containing the physical and optical properties of all LITT amenable tumors. These values could then be selected as lesion specific input prior to each procedure in order to allow for accurate real-time ablation estimates during LITT. In our cohort, residual tumor following ablation was noted in all five patients and presented as non-enhancing tumor core with a peripherally enhancing rim (Figure 1). All ablated WHO I meningiomas decreased in size despite the presence of a residual tumor, while the ablated high grade masses eventually progressed with recurrent tumor growth arising at the margins of the treated lesion (Figure 2). Incomplete ablation may be related to errors in ablation zone estimates, as discussed above. Another possible explanation is that Visualase laser probes (used in this study) release thermal energy in a symmetric spherical or cone shape pattern, which does not conform to the highly irregular shape of the EA masses leaving viable cells at the margins of the lesion. Neuroblate LITT platforms use a directional probe, which may be better suited for ablation of irregularly shaped lesions. Further developments in laser probe technology, as well as ablation zone estimates, may allow for more accurate ablation and potential improvements in progression-free survival in patients with high-grade EA tumors.

## Conclusions

In conclusion, our preliminary experience of five patients with EA meningiomas (four patients), and fibrous tumor (one patient) suggest that a successfully ablated lesion would have the following imaging characteristics: no significant change in size or configuration of the lesion in the initial post ablation periods, lack of gadolinium enhancement (vanishing enhancing sign) at the core of the ablated mass within the radius of effective thermal ablation (3 cm), with the lack of core enhancement being the most reliable imaging sign of successful treatment. Recurrence, if it occurs, will present at the periphery of the treated mass beyond the radius of effective thermal ablation.

## References

[1] Medvid R, Ruiz A., Komotar RJ (2015). Current applications of MRI-guided laser interstitial therapy in the treatment of brain neoplasms and epilepsy: a radiologic and neurosurgical overview. Am J Neuroradiol.

[2] Tatsui CE, Lee SH, Amini B (2016). Spinal laser interstitial thermal therapy: a novel alternative to surgery for metastatic epidural spinal cord compression. Neurosurgery.

[3] Kato A, Fujimoto Y, Taniguchi M (2004). Volumetric thermal devascularization of large meningiomas. J Neurosurg.

[4] Kato A, Fujimoto Y, Hashimoto N (2005). Radiofrequency thermal ablation for recurrent meningioma extending extracranially. Acta neurochirurgica.

[5] Jethwa PR, Barrese JC, Gowda A, Shetty A, Danish SF (2012). Magnetic resonance thermometry-guided laser-induced thermal therapy for intracranial neoplasms: initial experience. NEU.

[6] Ivan ME, Diaz RJ, Berger MH (2017). Magnetic resonance-guided laser ablation for the treatment of recurrent dural-based lesions: a series of five cases. World Neurosurg.

[7] Schober R, Bettag M, Sabel M, Ulrich F, Hessel S (1993). Fine structure of zonal changes in experimental Nd:YAG laser-induced interstitial hyperthermia. Lasers Surg Med.

[8] Schwabe B, Kahn T, Harth T, Ulrich F, Schwarzmaler HJ (1997). Laser-induced thermal lesions in the human brain: short-and long-term appearance on MRI. J Comput Assist Tomogr.

[9] Hawasli AH, Bagade S, Shimony JS, Miller-Thomas M, Leuthardt EC (2013). Magnetic resonance imaging-guided focused laser interstitial thermal therapy for intracranial lesions: single-institution series. Neurosurgery.

[10] Jethwa PR, Lee JH, Assina R, Keller IA, Danish SF (2011). Treatment of a supratentorial primitive neuroectodermal tumor using magnetic resonance-guided laser-induced thermal therapy: technical note. J Neurosurg Pediatr.

[11] Eggert HR, Blazek V (1987). Optical properties of human brain tissue, meninges, and brain tumors in the spectral range of 200 to 900 nm. Neurosurgery.

